# {4,4′,6,6′-Tetra­bromo-2,2′-[2,2-dimethyl­propane-1,3-diylbis(nitrilo­methanylyl­idene)]diphenolato}nickel(II)

**DOI:** 10.1107/S1600536812020375

**Published:** 2012-05-12

**Authors:** Hadi Kargar, Reza Kia, Muhammad Nawaz Tahir

**Affiliations:** a Department of Chemistry, Payame Noor University, PO Box 19395-3697 Tehran, I. R. of IRAN; b Department of Chemistry, Science and Research Branch, Islamic Azad University, Tehran, Iran; c Department of Physics, University of Sargodha, Punjab, Pakistan

## Abstract

The asymmetric unit of the title compound, [Ni(C_19_H_16_Br_4_N_2_O_2_)], comprises half of a Schiff base complex. The geometry around the Ni^II^ atom, located on a twofold rotation axis, is distorted square-planar, which is supported by the N_2_O_2_ donor atoms of the coordinated ligand. The dihedral angle between the substituted benzene rings is 23.19 (17)°. In the crystal, a short inter­molecular Br⋯Br [3.6475 (7) Å] inter­action is present.

## Related literature

For applications of Schiff bases in coordination chemistry, see: Granovski *et al.* (1993 ▶); Blower (1998 ▶). For related structures, see: Ghaemi *et al.* (2011 ▶); Kargar *et al.* (2012 ▶). For van der Waals radii, see: Bondi (1964 ▶). For standard values of bond lengths, see: Allen *et al.* (1987 ▶).
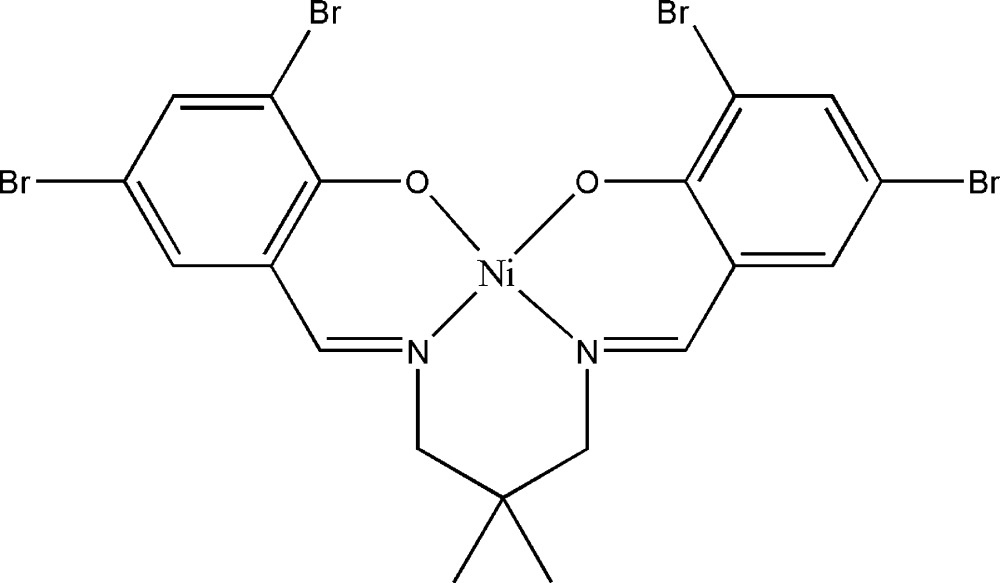


## Experimental

### 

#### Crystal data

[Ni(C_19_H_16_Br_4_N_2_O_2_)]
*M* *_r_* = 682.69
Orthorhombic, 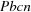

*a* = 16.1125 (11) Å
*b* = 15.4789 (12) Å
*c* = 8.4734 (5) Å
*V* = 2113.3 (3) Å^3^
*Z* = 4
Mo *K*α radiation
μ = 8.50 mm^−1^
*T* = 296 K
0.25 × 0.18 × 0.09 mm

#### Data collection

Bruker SMART APEXII CCD area-detector diffractometer
Absorption correction: multi-scan (*SADABS*; Bruker, 2005 ▶) *T*_min_ = 0.694, *T*_max_ = 0.871
16236 measured reflections
2086 independent reflections
1574 reflections with *I* > 2σ(*I*)
*R*_int_ = 0.049

#### Refinement

*R*[*F* ^2^ > 2σ(*F* ^2^)] = 0.033
*wR*(*F* ^2^) = 0.075
*S* = 1.05
2086 reflections
129 parameters
H-atom parameters constrained
Δρ_max_ = 0.52 e Å^−3^
Δρ_min_ = −0.78 e Å^−3^

### 

Data collection: *APEX2* (Bruker, 2005 ▶); cell refinement: *SAINT* (Bruker, 2005 ▶); data reduction: *SAINT*; program(s) used to solve structure: *SHELXS97* (Sheldrick, 2008 ▶); program(s) used to refine structure: *SHELXL97* (Sheldrick, 2008 ▶); molecular graphics: *SHELXTL* (Sheldrick, 2008 ▶); software used to prepare material for publication: *SHELXTL* and *PLATON* (Spek, 2009 ▶).

## Supplementary Material

Crystal structure: contains datablock(s) global, I. DOI: 10.1107/S1600536812020375/su2421sup1.cif

Structure factors: contains datablock(s) I. DOI: 10.1107/S1600536812020375/su2421Isup2.hkl

Additional supplementary materials:  crystallographic information; 3D view; checkCIF report

## Figures and Tables

**Fig. 1.**
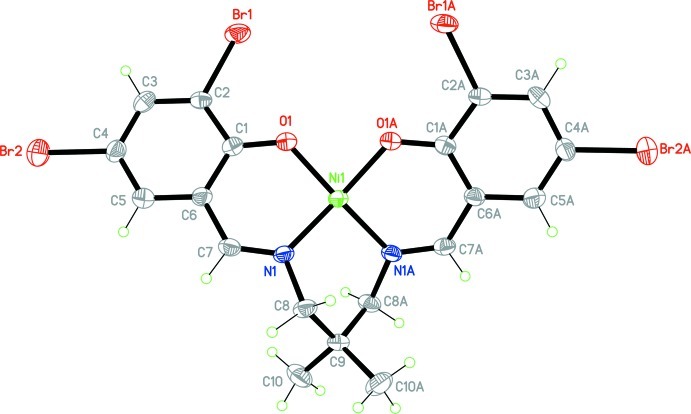
A view of the molecular structure of the title compound, showing 40% probability displacement ellipsoids and the atomic numbering [symmetry code: (A) = -x, y, -z+1/2].

**Fig. 2.**
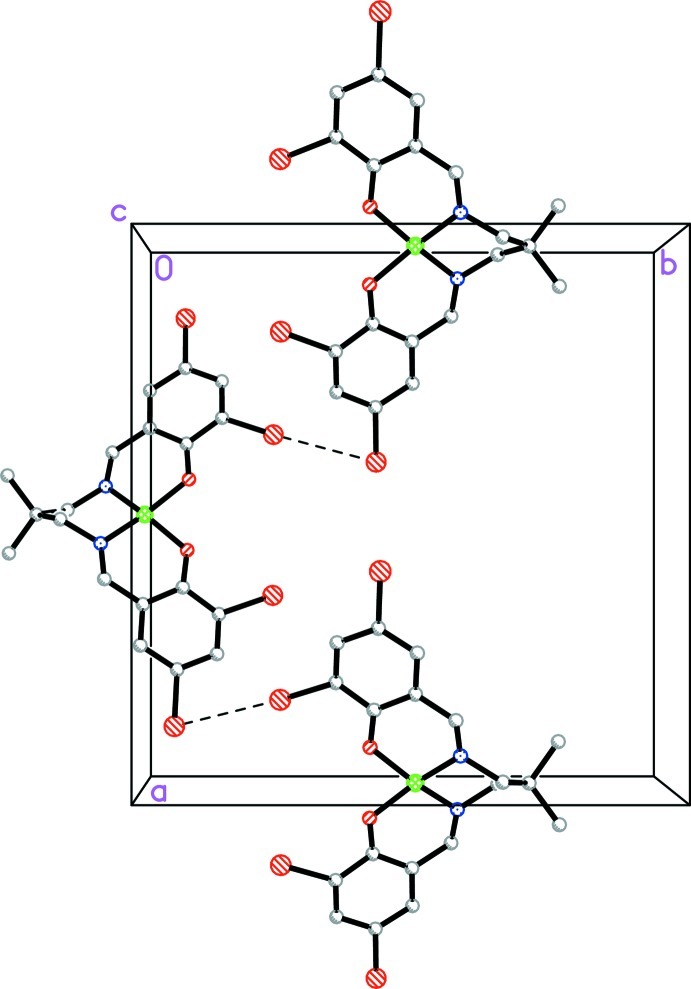
The crystal packing of the title compound viewed along the c-axis, showing how the molecules are linked via the intermolecular Br···Br interactions (dashed lines) to form chains along the a axis [the H atoms have been omitted for clarity].

## supplementary crystallographic information

### Comment

Schiff base complexes are one of the most important stereochemical models in transition metal coordination chemistry, with ease of preparation and structural variations (Granovski *et al.*, 1993; Blower *et al.*, (1998). In continuation of our work on the crystal structures of Schiff base metal complexes (Kargar *et al.*, 2012; Ghaemi *et al.*, 2011), we report herein on the crystal structure of the title compound.

The asymmetric unit of the title compound, Fig. 1, comprises half of a Schiff base complex. The nickel(II) atom and the central bridging C atom, C9, are located on a 2-fold rotation axis. The bond lengths (Allen *et al.*, 1987) and angles are within normal ranges and are comparable to those reported for related structures (Kargar *et al.*, 2012; Ghaemi *et al.*, 2011).

The geometry around Ni^II^ is a distorted square-planar which is supported by the N_2_O_2_ donor atoms of the coordinated Schiff base ligand. The dihedral angle between the substituted benzene rings is 23.19 (17)°.

In the crystal (Fig. 2), a short intermolecular Br1···Br2^i^ [3.6475 (7)Å; symmetry code: (i) = -*x*+1/2, -*y*-1/2, *z*+1/2] interaction is present, which is shorter than the sum of the van der Waals radius of Br atoms [3.70 Å; Bondi, 1964].

### Experimental

The title compound was synthesized by adding 3,5-dibromo-salicylaldehyde-2,2-dimethyl-1, 3-propanediamine (2 mmol) to a solution of NiCl_2_. 6H_2_O (2.1 mmol) in ethanol (30 ml). The mixture was refluxed with stirring for half an hour. The resultant solution was filtered. Dark-green single crystals of the title compound suitable for *X*-ray structure determination were recrystallized from ethanol by slow evaporation of the solvents at room temperature over several days.

### Refinement

The H-atoms were included in calculated positions and treated as riding atoms: C—H = 0.93, 0.96 and 0.97 Å for CH, CH_3_ and CH_2_ H-atoms, respectively, with U_iso_ (H) = k x U_eq_(C), where k = 1.5 for CH_3_ H-atoms, and = 1.2 for other H-atoms.

### Crystal data

**Table:**

[Ni(C_19_H_16_Br_4_N_2_O_2_)]	*F*(000) = 1312
*M**_r_* = 682.69	*D*_x_ = 2.146 Mg m^−^^3^
Orthorhombic, *P**b**c**n*	Mo *K*α radiation, λ = 0.71073 Å
Hall symbol: -P 2n 2ab	Cell parameters from 2540 reflections
*a* = 16.1125 (11) Å	θ = 2.5–27.4°
*b* = 15.4789 (12) Å	µ = 8.50 mm^−^^1^
*c* = 8.4734 (5) Å	*T* = 296 K
*V* = 2113.3 (3) Å^3^	Block, dark-red
*Z* = 4	0.25 × 0.18 × 0.09 mm

### Data collection

**Table:**

Bruker SMART APEXII CCD area-detector diffractometer	2086 independent reflections
Radiation source: fine-focus sealed tube	1574 reflections with *I* > 2σ(*I*)
Graphite monochromator	*R*_int_ = 0.049
φ and ω scans	θ_max_ = 26.0°, θ_min_ = 1.8°
Absorption correction: multi-scan (*SADABS*; Bruker, 2005)	*h* = −19→19
*T*_min_ = 0.694, *T*_max_ = 0.871	*k* = −14→19
16236 measured reflections	*l* = −10→10

### Refinement

**Table:**

Refinement on *F*^2^	Primary atom site location: structure-invariant direct methods
Least-squares matrix: full	Secondary atom site location: difference Fourier map
*R*[*F*^2^ > 2σ(*F*^2^)] = 0.033	Hydrogen site location: inferred from neighbouring sites
*wR*(*F*^2^) = 0.075	H-atom parameters constrained
*S* = 1.05	*w* = 1/[σ^2^(*F*_o_^2^) + (0.0387*P*)^2^ + 0.4809*P*] where *P* = (*F*_o_^2^ + 2*F*_c_^2^)/3
2086 reflections	(Δ/σ)_max_ = 0.001
129 parameters	Δρ_max_ = 0.52 e Å^−^^3^
0 restraints	Δρ_min_ = −0.78 e Å^−^^3^

### Special details

**Table:**

Geometry. All esds (except the esd in the dihedral angle between two l.s. planes) are estimated using the full covariance matrix. The cell esds are taken into account individually in the estimation of esds in distances, angles and torsion angles; correlations between esds in cell parameters are only used when they are defined by crystal symmetry. An approximate (isotropic) treatment of cell esds is used for estimating esds involving l.s. planes.
Refinement. Refinement of F^2^ against ALL reflections. The weighted R-factor wR and goodness of fit S are based on F^2^, conventional R-factors R are based on F, with F set to zero for negative F^2^. The threshold expression of F^2^ > 2sigma(F^2^) is used only for calculating R-factors(gt) etc. and is not relevant to the choice of reflections for refinement. R-factors based on F^2^ are statistically about twice as large as those based on F, and R- factors based on ALL data will be even larger.

### Fractional atomic coordinates and isotropic or equivalent isotropic displacement parameters (Å^2^)

**Table:**

	*x*	*y*	*z*	*U*_iso_*/*U*_eq_	
Br1	0.34274 (3)	−0.24016 (2)	0.18392 (5)	0.04218 (15)	
Br2	0.10156 (3)	−0.05088 (3)	−0.14763 (7)	0.06551 (19)	
Ni1	0.5000	0.02131 (4)	0.2500	0.02663 (17)	
O1	0.43065 (15)	−0.06700 (15)	0.1850 (3)	0.0344 (6)	
N1	0.44411 (18)	0.10588 (18)	0.1331 (3)	0.0281 (7)	
C1	0.3595 (2)	−0.0600 (2)	0.1155 (4)	0.0284 (8)	
C2	0.3081 (2)	−0.1331 (2)	0.0964 (4)	0.0293 (8)	
C3	0.2334 (2)	−0.1309 (3)	0.0186 (4)	0.0337 (9)	
H3	0.2019	−0.1809	0.0073	0.040*	
C4	0.2054 (2)	−0.0533 (3)	−0.0427 (4)	0.0372 (9)	
C5	0.2510 (2)	0.0203 (3)	−0.0270 (5)	0.0391 (10)	
H5	0.2312	0.0721	−0.0681	0.047*	
C6	0.3279 (2)	0.0180 (2)	0.0515 (4)	0.0304 (8)	
C7	0.3763 (2)	0.0952 (2)	0.0563 (4)	0.0317 (8)	
H7	0.3569	0.1420	−0.0017	0.038*	
C8	0.4910 (2)	0.1859 (2)	0.1043 (4)	0.0334 (9)	
H8A	0.4631	0.2189	0.0227	0.040*	
H8B	0.5458	0.1711	0.0655	0.040*	
C9	0.5000	0.2419 (3)	0.2500	0.0320 (12)	
C10	0.4239 (3)	0.2995 (3)	0.2735 (5)	0.0499 (11)	
H10A	0.3746	0.2647	0.2718	0.075*	
H10B	0.4213	0.3415	0.1901	0.075*	
H10C	0.4280	0.3286	0.3732	0.075*	

### Atomic displacement parameters (Å^2^)

**Table:**

	*U*^11^	*U*^22^	*U*^33^	*U*^12^	*U*^13^	*U*^23^
Br1	0.0576 (3)	0.0265 (2)	0.0424 (3)	−0.00786 (19)	−0.00041 (19)	0.00544 (17)
Br2	0.0449 (3)	0.0558 (3)	0.0958 (4)	−0.0040 (2)	−0.0292 (3)	−0.0020 (3)
Ni1	0.0301 (3)	0.0205 (4)	0.0292 (3)	0.000	−0.0007 (3)	0.000
O1	0.0369 (15)	0.0238 (14)	0.0425 (14)	−0.0025 (12)	−0.0059 (12)	0.0023 (12)
N1	0.0348 (17)	0.0206 (16)	0.0290 (16)	−0.0047 (14)	−0.0020 (13)	0.0011 (13)
C1	0.033 (2)	0.027 (2)	0.0257 (18)	−0.0035 (17)	0.0038 (16)	−0.0003 (16)
C2	0.036 (2)	0.0226 (19)	0.0293 (19)	−0.0016 (16)	0.0045 (16)	0.0015 (16)
C3	0.033 (2)	0.035 (2)	0.033 (2)	−0.0105 (18)	0.0043 (16)	−0.0045 (18)
C4	0.030 (2)	0.038 (2)	0.044 (2)	−0.0040 (19)	−0.0042 (17)	−0.0042 (19)
C5	0.043 (2)	0.031 (2)	0.043 (2)	0.0032 (19)	−0.0058 (18)	−0.0022 (19)
C6	0.034 (2)	0.026 (2)	0.0315 (19)	−0.0034 (16)	−0.0011 (16)	0.0023 (16)
C7	0.040 (2)	0.023 (2)	0.0326 (19)	0.0003 (17)	0.0001 (17)	0.0042 (17)
C8	0.045 (2)	0.024 (2)	0.0313 (19)	−0.0084 (18)	−0.0001 (17)	0.0036 (16)
C9	0.038 (3)	0.020 (3)	0.038 (3)	0.000	−0.001 (2)	0.000
C10	0.057 (3)	0.040 (3)	0.053 (3)	0.019 (2)	−0.006 (2)	0.003 (2)

### Geometric parameters (Å, º)

**Table:**

Br1—C2	1.899 (3)	C4—C5	1.362 (5)
Br2—C4	1.895 (4)	C5—C6	1.407 (5)
Ni1—O1	1.849 (2)	C5—H5	0.9300
Ni1—O1^i^	1.849 (2)	C6—C7	1.428 (5)
Ni1—N1^i^	1.872 (3)	C7—H7	0.9300
Ni1—N1	1.872 (3)	C8—C9	1.515 (4)
O1—C1	1.293 (4)	C8—H8A	0.9700
N1—C7	1.282 (4)	C8—H8B	0.9700
N1—C8	1.471 (4)	C9—C8^i^	1.515 (4)
C1—C2	1.412 (5)	C9—C10^i^	1.529 (5)
C1—C6	1.418 (5)	C9—C10	1.529 (5)
C2—C3	1.373 (5)	C10—H10A	0.9600
C3—C4	1.384 (5)	C10—H10B	0.9600
C3—H3	0.9300	C10—H10C	0.9600
			
O1—Ni1—O1^i^	84.69 (15)	C5—C6—C1	121.3 (3)
O1—Ni1—N1^i^	164.78 (11)	C5—C6—C7	118.3 (3)
O1^i^—Ni1—N1^i^	93.94 (11)	C1—C6—C7	120.3 (3)
O1—Ni1—N1	93.94 (11)	N1—C7—C6	126.0 (3)
O1^i^—Ni1—N1	164.78 (11)	N1—C7—H7	117.0
N1^i^—Ni1—N1	91.27 (17)	C6—C7—H7	117.0
C1—O1—Ni1	127.5 (2)	N1—C8—C9	113.3 (3)
C7—N1—C8	117.5 (3)	N1—C8—H8A	108.9
C7—N1—Ni1	126.0 (3)	C9—C8—H8A	108.9
C8—N1—Ni1	115.5 (2)	N1—C8—H8B	108.9
O1—C1—C2	120.4 (3)	C9—C8—H8B	108.9
O1—C1—C6	124.4 (3)	H8A—C8—H8B	107.7
C2—C1—C6	115.3 (3)	C8—C9—C8^i^	110.2 (4)
C3—C2—C1	123.3 (3)	C8—C9—C10^i^	107.7 (2)
C3—C2—Br1	117.8 (3)	C8^i^—C9—C10^i^	111.3 (2)
C1—C2—Br1	118.8 (3)	C8—C9—C10	111.3 (2)
C2—C3—C4	119.2 (3)	C8^i^—C9—C10	107.7 (2)
C2—C3—H3	120.4	C10^i^—C9—C10	108.7 (5)
C4—C3—H3	120.4	C9—C10—H10A	109.5
C5—C4—C3	120.9 (3)	C9—C10—H10B	109.5
C5—C4—Br2	120.4 (3)	H10A—C10—H10B	109.5
C3—C4—Br2	118.7 (3)	C9—C10—H10C	109.5
C4—C5—C6	120.0 (4)	H10A—C10—H10C	109.5
C4—C5—H5	120.0	H10B—C10—H10C	109.5
C6—C5—H5	120.0		
			
O1^i^—Ni1—O1—C1	−178.8 (3)	C2—C3—C4—Br2	−179.3 (3)
N1^i^—Ni1—O1—C1	95.7 (5)	C3—C4—C5—C6	0.5 (6)
N1—Ni1—O1—C1	−14.0 (3)	Br2—C4—C5—C6	179.8 (3)
O1—Ni1—N1—C7	6.6 (3)	C4—C5—C6—C1	−0.1 (5)
O1^i^—Ni1—N1—C7	90.8 (5)	C4—C5—C6—C7	175.9 (3)
N1^i^—Ni1—N1—C7	−159.1 (4)	O1—C1—C6—C5	178.0 (3)
O1—Ni1—N1—C8	−161.3 (2)	C2—C1—C6—C5	−0.9 (5)
O1^i^—Ni1—N1—C8	−77.1 (5)	O1—C1—C6—C7	2.2 (5)
N1^i^—Ni1—N1—C8	32.97 (18)	C2—C1—C6—C7	−176.7 (3)
Ni1—O1—C1—C2	−169.7 (2)	C8—N1—C7—C6	171.2 (3)
Ni1—O1—C1—C6	11.5 (5)	Ni1—N1—C7—C6	3.5 (5)
O1—C1—C2—C3	−177.4 (3)	C5—C6—C7—N1	174.0 (3)
C6—C1—C2—C3	1.5 (5)	C1—C6—C7—N1	−10.0 (6)
O1—C1—C2—Br1	3.1 (4)	C7—N1—C8—C9	118.4 (3)
C6—C1—C2—Br1	−177.9 (2)	Ni1—N1—C8—C9	−72.7 (3)
C1—C2—C3—C4	−1.1 (5)	N1—C8—C9—C8^i^	35.2 (2)
Br1—C2—C3—C4	178.3 (3)	N1—C8—C9—C10^i^	156.7 (3)
C2—C3—C4—C5	0.1 (6)	N1—C8—C9—C10	−84.3 (4)

Symmetry code: (i) −*x*+1, *y*, −*z*+1/2.
